# Group exercise in long-term care facilities, alignment with World Health Organization recommendations: a cross-sectional survey

**DOI:** 10.1007/s40520-025-02954-4

**Published:** 2025-02-22

**Authors:** Salud Poveda-López, Carmen Lillo-Navarro, Joaquina Montilla-Herrador

**Affiliations:** 1 Faculty of Physiotherapy, Podiatry and Occupational Therapy, UCAM Catholic University of Murcia, Murcia, Spain; 2 Department of Pathology and Surgery, Center for Translational Research in Physiotherapy (CEIT), University Miguel Hernández, Sant Joan, Alicante, Spain; 3 CEIR Campus Mare Nostrum (CMN), University of Murcia, Instituto Murciano de Investigación Biosanitaria-Virgen de la Arrixaca (IMIB-Arrixaca), El Palmar, Murcia, Spain

**Keywords:** Exercise, Evidence-based practice, Long-term care, Aged, Older population

## Abstract

**Background:**

Maintaining functional status in institutionalized older people is a challenge for long-term care (LTC) institutions. In this regard, exercise may have positive effects. The World Health Organization (WHO) has issued guidelines which include recommendations of exercise for each population group. Nonetheless, the literature shows that the levels of exercise among institutionalized population are still low.

**Aims:**

This study sought to determine: (1) the characteristics of exercise programs for older people performed by health professionals in LTC facilities, (2) the knowledge and use of the WHO recommendations and guidelines for exercising among older people in LTC facilities; (3) the limitations identified by health professionals regarding the application of the WHO guidelines.

**Materials and methods:**

A cross-sectional national survey following STROBE guideline was performed. Sample: professionals developing exercise programs for institutionalized older people. A Delphi study was conducted to create the survey which included sociodemographic data, exercise characteristics, knowledge about WHO recommendations and limitations regarding their application. Descriptive statistics were used on the data, such as Pearson’s χ2 and independent t- test.

**Results:**

Many professionals do not know (27,5%) or do not follow (52%) the guidelines proposed by the WHO. There is a low weekly frequency for strength exercises (30%) and aerobic exercise (51%). The professional contract influences the weekly frequency of exercise. Most identified limitations for using the WHO recommendations were the lack of time and large groups.

**Discussion and conclusions:**

Recommendations of WHO guidelines are familiar to many professionals, however, some are difficult to implement in exercise programs in LTC facilities.

## Introduction

The rise in life expectancy is accompanied by an increase in the number of people suffering from chronic diseases [1, 2]. Dependence has also been growing, and the affected individuals usually require care and assistance from a third person. Consequently, the institutionalization among this population is on the rise, as families are often unable to take care of them [3, 4]. Older people living in institutions usually have numerous pathologies, they are often polymedicated, and their complex situation makes them highly dependent on activities of daily living [5].

One of the challenges for health professionals working with institutionalized older people is to maintain or improve the functional capacity of the users as long as possible. Overall physical activity and exercise have shown to protect against disabilities and their progression in the general population [6, 7]. Indeed, the effects of physical activity and exercise are well documented [8, 9] and can help to prevent certain heart diseases [10], sarcopenia [11], osteoporosis and other musculoskeletal conditions and even improve mental disorders, such as depression [12] or dementia [13], which are highly prevalent diseases in older people. In addition, experts on institutionalized older people, together with other organisations such as the World Health Organization (WHO) [14, 15] or the International Association of Gerontology and Geriatrics, have recognized the importance of exercise for this population group to improve their quality of life [16]. Furthermore, the evidence-based literature has shown that exercising has positive effects on the functional capacity to perform activities of daily living among institutionalized older people [17, 18].

For these reasons, institutions should offer fully individualized care and strive towards improving the quality of life of users [19]. However, due to the heterogeneity of this population, together with the comorbidities and decreased independence, the implementation of exercise recommendations is very difficult. Indeed, the levels of physical activity and exercise, leisure activities and health status are still low among the institutionalized population [20].

Some studies suggest that institutionalized older people may benefit from a comprehensive program of stimulation including both physical and mental domains directed by a transdisciplinary team [21]. Thus, physiotherapists and occupational therapists appear to be the most suitable professionals to organize and direct the physical activity and the exercise programs [22].

Evidence-based practice is essential in the health care setting, as it supports the quality of patient care. Several evidence-based exercise guidelines state the amount and the type of exercise that should be performed for the well-being of the older population [14, 23]; however, the current guidelines on physical activity and exercise for older people are more appropriate for community dwelling older people than for those living in institutions [24]. Moreover, evidence-based research and guidelines are not always implemented in clinical practice [25, 26]. In this regard, although the literature has previously identified several barriers to implementing the guidelines in clinical practice in certain qualitative designs [27], it is still unknown whether the professionals who direct the exercise in LTC institutions are aware of the guidelines or whether they implement them.

The aims of this study were: (1) to determine the characteristics of exercise programs for older people carried out by health professionals in LTC facilities, (2) To determine whether professionals know and use the WHO exercise recommendations and guidelines for older people in LTC facilities; (3) to understand the limitations identified by health professionals when applying the WHO guidelines in clinical practice.

## Methods

### Study design

A descriptive research design following the Strengthening the Reporting of Observational Studies in Epidemiology (STROBE) [28] statement was carried out to investigate the development of exercise programs in LTC institutions. A cross-sectional survey was conducted.

### Sample

The inclusion criteria were professionals responsible for implementing exercise programs for older people in LTC institutions in Spain who were working or had worked for at least six months over the last five years in LTC institutions for older people. The survey was distributed nationwide.

### Survey development

Since no existing survey met the requirements of this study, a specially designed survey was created, reviewed, and implemented. To ensure face and content validity, the authors developed the questions after reviewing the evidence on pediatric physiotherapy and engaging in extensive discussions with an expert group using the Delphi methodology [29]. The Delphi technique is often used to obtain an informed or refined consensus from a group of knowledgeable experts or informants. A three-round survey was done. Participants were given one week to complete each round and after receiving the responses, the researchers analyzed the data for one week. Following the results of the first round, a second survey was generated and returned to the experts. This procedure was repeated until consensus was reached, after the third round. An 80% agreement among the experts was deemed necessary, both in terms of content and relevance, representativeness, and sufficiency.

Fourteen physiotherapists from different parts of Spain and working in the field of geriatric physiotherapy in nursing homes were selected. They were contacted by phone and email to request their participation. All prospective participants were informed about the study procedures and informed consent to participate was requested. After agreeing to participate in the study, each expert was assigned a code to facilitate anonymity among the researchers. Reminder emails were sent after each phase. Ultimately, only 10 participants completed the study.

### Survey dissemination

The survey generated after the Delphi process was disseminated throughout Spain. It was sent by email to professional associations, spread through organizations, and sent to the different LTC facilities in Spain. A self-administered online survey was completed by the participants.

### Ethical considerations

Ethical approval for this study was granted by the Research Ethics Board at the University of Murcia, Spain. The survey was anonymous and no personal data such as name, e-mail address or name of the institution was collected.

On the main page of the survey, the participants received all the information regarding the study, the endorsement of the ethics committee and the informed consent.

### Measurements

The survey consisted of three parts: (1) Socio-demographic information and professional data, (2) Data regarding the facility organization and exercise program characteristics and (3) knowledge, follow-up, and barriers in applying recommendations from the WHO guidelines to exercise programs.

The final version of the (37)-question survey was posted on Google forms for online access.

#### Variables: sociodemographic and professional data

The sociodemographic data was gathered based on 7 questions related to gender, age, qualification, training and work experience with older people.

The variable representing the number of weekly working hours of the professionals was recorded to distinguish between professionals with full-time contracts (≥ 35 h/week) and those with part-time contracts (< 35 h/week).

#### Variables: characteristics of exercise programs

The information of the characteristics of exercise programs was collected in 14 questions including: number of residents of the LTC institution, how the exercise program was conducted (individual or groups), hours per week spent on exercise programs, number of groups in the institution, number of residents per group, groups organization (separated or not according to residents’ functional or cognitive status), position in which residents perform the exercise, exercise guidance and development of exercise programs.

#### Variables: types of exercises performed

This information was obtained through 15 questions. We used the WHO guidelines on physical activity and sedentary behavior (WHO guideline, 2020). These recommendations include three overall types of activities (aerobic activity, muscle-strengthening activity, and multicomponent exercise). Respondents were asked to rate the presence of these activities in exercise programs using dichotomous response categories (yes/no). Additionally, professionals’ perception of their knowledge about the WHO guidelines was assessed with a dichotomous question. Follow-up on adherence to the WHO guidelines was investigated with a question offering five response options.

A question was also included regarding the types of exercise, as described by the WHO guidelines: aerobic activity, strength exercise and balance exercise. Additionally, there were questions about other multi-component physical activities performed beyond the exercise program, such as walking, dancing, games, and outings.

For the exercise activities, respondents were asked about dosage: weekly frequency, number of exercises and the number of repetitions. Information about other types of exercise (mobility, coordination, flexibility, respiratory exercises) which were not included in the guidelines was also requested.

#### Variable: barriers in using or knowing the WHO guidelines

We collected information about the barriers and limitations founded by the participants in using or knowing the WHO guidelines through one question of the survey.

### Statistical analysis

We used descriptive statistics, including Pearson χ2 and independent t-tests, to summarize variables regarding the three domains between professionals with adherence and without adherence with all types of exercise proposed in the WHO guidelines.

Relationships between different variables were then assessed through contingency tables leading into Chi-squared tests or Fisher exact tests, depending on the percentage of expected frequencies lower than 5.

All analyses were performed using IBM SPSS Statistics for Windows, Version 28.0 (Armonk, NY, USA: IBM Corp; 2016), with a p-level of significance set at *p* < 0.05.

## Results

### Characteristics of participants

A total of 200 people participated, 138 women and 62 men aged between 21 and 60 years old (35.83 ± 8.78). Of these, 177 were physiotherapists and 23 were other employees of the institution, such as occupational therapists, psychologists, nurses and graduates in physical activity and sports. The participants had completed their bachelor’s studies, and 58 of them (29%) had postgraduate studies.

In terms of the experience of the participants working in LTC institutions with older people, 43% had over 10 years of experience, 19% had 5–9 years’ experience, and 38% had less than 5 years’ experience.

Most of the respondents (72,5%) stated that they were aware of the WHO guidelines. The characteristics of the participants are shown in Table 1.

**Table 1:** Socio-demographic and professionals characteristics of the participants

Variables (*n* = 200)		*N* (%)		
Gender	Female		138 (69%)	
	Male		62 (31%)	
Age (years)	21–30		66 (33%)	
	31–45		104 (52%)	
	46 or more		30 (15%)	
Qualification	Physiotherapist		177 (88,5%)	
	Another one		23 (11,5%)	
Training	Bachelor		142 (71%)	
	Postgraduate		58 (29%)	
Experience working in LTC institutions	Under 5 years		76 (38%)	
	5 to 9 years		38 (19%)	
	10 years or more		86 (43%)	
Knowledge of the WHO guidelines	Yes		145 (72,5%)	
	No		55 (27,5%)	

### Organization and characteristics of the exercise programs

The number of residents in the institutions varied. Some small centres had 30–50 users, whereas others had over 100 users. Participants working in larger centers were more familiar with the WHO guidelines than those working in smaller centers (p 0.029). 

**Table 2:** Facility organization factors and exercise program characteristics associated with lack of knowledge or non-use of WHO guidelines

Variables		All professionals *N* (%)	Professionals who do not know WHO guidelines *N* (%)	P*	Professionals who do not use WHO guidelines *N* (%)	P**
		(*n* = 200)	(*n* = 55)		(*n* = 104)	
Number of residents in the LTC facility	Between 30–50	53 (26,5%)	22 (40%)	*0*,029***	31 (29,8%)	0,359
	Between 51–100	70 (35%)	16 (29,1%)		32 (30,8%)	
	More than 100	77 (38,5%)	17 (30,9%)		41 (39,4%)	
Conducting exercise programs at the LTC facility	In groups	15 (7,5%)	5 (9,1%)	0,819	8 (7,7%)	
	In groups and individual	179 (89,5)	48 (87,3%)		92 (88,5%)	0,758
	Only individual	6 (3%)	2 (3,6%)		4 (3,8%)	
		**(** ***n*** ** = 194)**	**(** ***n*** ** = 53)**		**(** ***n*** ** = 100)**	
Hours per week spent on exercise programs	Less than 3 h per week	70 (36%)	23 (43,4%)	*0*,006***	45 (45%)	*0*,006***
	Between 4–5 h per week	89 (45,9%)	28 (52,8%)		44 (44%)	
	6 or more hours per week	35 (18,1%)	2 (3,8%)		11 (11%)	
Number of exercise groups in the LTC facility	Between 1 and 2	92 (47,4%)	29 (54,7%)	0,443	53 (53%)	0,272
	Between 3 and 4	62 (32%)	14 (26,4%)		29 (29%)	
	5 or more	40 (20,6%)	10 (18,9%)		18 (18%)	
Number of residents per exercise group	Less than 10	47 (24,2%)	12 (22,6%)	0,666	21 (21%)	0,582
	10 to 20	100 (51,6%)	26 (49%)		53 (53%)	
	More than 20	47 (24,2%)	15 (28,3%)		26 (26%)	
Groups organized separated by functional capacity of the residents	Yes	116 (59,8%)	27 (50,9%)	0,123	56 (56%)	0,266
	No	78 (40,2%)	26 (49,1%)		44 (44%)	
Opportunity to participate in exercise programs according to residents’ functional status.	All users without exception can participate	32 (16,5%)	9 (17%)	0,682	13 (13%)	0,398
	All users except bedridden users can participate	160 (82,5%)	44 (83%)		86 (86%)	
	Only those who can move independently can participate	2 (1%)	0 (0%)		1 (1%)	
Groups organized separated by cognitive status of the residents	Yes	122 (62,9%)	32 (60,4%)	0,657	60 (60%)	0,391
	No	72 (37,1%)	21 (39,6%)		40 (40%)	
Opportunity to participate in exercise programs according to residents’ cognitive status.	All users without exception can participate	79 (40,7%)	24 (45,3%)	0,212	38 (38%)	0,599
	Those with no impairment and those with mild impairment can participate.	19 (9,8%)	2 (3,8%)		9 (9%)	
	Those with no impairment and those with mild and moderate impairment can participate	96 (49,5%)	27 (50,9%)		53 (53%)	
	Only can participate users with no cognitive impairment	0 (0%)	0 (0%)		0 (0%)	
Position in which residents perform exercise program	Seated	53 (27,3%)	20 (37,7%)	0,136	36 (36%)	*0*,018***
	Standing	0 (0%)	0 (0%)		0 (0%)	
	Some in seated and some in standing position depending on functional capacity.	85 (43,8%)	20 (37,7%)		40 (40%)	
	In both positions depending on the exercise	56 (28,9%)	13 (24,6%)		24 (24%)	
Professional who directs the exercise program	Physiotherapist	176 (90,7%)	48 (90,6%)	0,963	90 (90%)	0,721
	Another one	18(9,3%)	5 (9,4%)		10 (10%)	
Number of professionals supervising the exercise program	1	139 (71,6%)	44 (83%)	0,031***	79 (79%)	0,019***
	More than 1	55 (28,4%)	9 (17%)		21 (21%)	
What professionals develop the exercise programs	Exclusively the professional directing the activity	106 (54,6%)	36 (67,9%)	0,058	56 (56%)	0,356
	The one who leads the activity and other professionals of the team	84 (43,3%)	16 (30,2%)		42 (42%)	
	The programs are elaborated by other professionals and directed by someone else	4 (2,1%)	1 (1,9%)		2 (2%)	
Decision-making capacity of the professional directing the exercise programs	Low decision-making capacity	70 (36,1%)	19 (35,8%)	0,967	33 (33%)	0,356
	High decision-making capacity	124 (63,9%)	34 (64,2%)		67 (67%)	

^* p value of the differences between professionals who know vs. those who do not know WHO guidelines ** p value of the differences between professionals who use vs. those who do not use WHO guidelines *** *p* < 0.05^

Most of the professionals working in institutions performed both group and individual exercise and only six professionals indicated that they worked in institutions where there was no group exercise program, performing only individual exercise programs or sessions.

In general, the weekly time devoted to group exercise sessions was between four and five hours per week. About 36% of respondents spent less than three hours per week and very few (18,1%) spent more than six hours per week. These findings seem to be related to both variables: thus, participants who did not know the WHO guidelines (p 0.006) and those who stated that they failed to apply them in their exercise programs (p 0.006) spent less time carrying out the exercise programs.

Among the professionals who performed group exercises in their institution, most of them organized the residents in one or two exercise groups. In contrast, only 20,6% of them organized five or more groups. Furthermore, there was a wide range of number of users per group, from less than 10 (24,2%) to more than 20 (24,2%) users per group.

The position in which the residents performed the exercises varied depending on the institution. Some professionals reported that residents only exercised in a seated position, however, a high percentage of participants (43,8%) claimed that residents exercised both in a seated and standing position, depending on their functional status. Additionally, 28,9% exercised in both positions depending on the exercise being performed.

A significant percentage of professionals (27,3%) exclusively performed the exercise program in sitting. This percentage increased to 36% among those who reported not using the WHO guidelines (*p* = 0.004). The position in which the participants performed the exercise programs was related to participants who did not implement the guidelines in their exercise programs (*p* = 0.018).

Group supervision was mainly provided by a single health professional (71,6%), who was usually a physiotherapist (90,7%). The fact that the groups were supervised by a single professional appeared to be relevant in terms of knowledge of the WHO guidelines and their implementation during the development of the exercise programs, although it was not statistically significant.

Groups were often organized based on the functional and cognitive capacities of the residents. The opportunity to participate in exercise programs was provided according to residents’ functional and cognitive status. Regarding the functional status, 82,5% of respondents stated that all users could participate except those who were bedridden. Concerning the cognitive status, 40,7% of participants claimed that there was no restriction and 59,3% responded that users with moderate impairment could participate.

One third of professionals developing exercise programs considered that they had limited capacity to decide the components of the exercise program in their institutions (See Table 2).

### Type of exercise performed

**Fig. 1:**
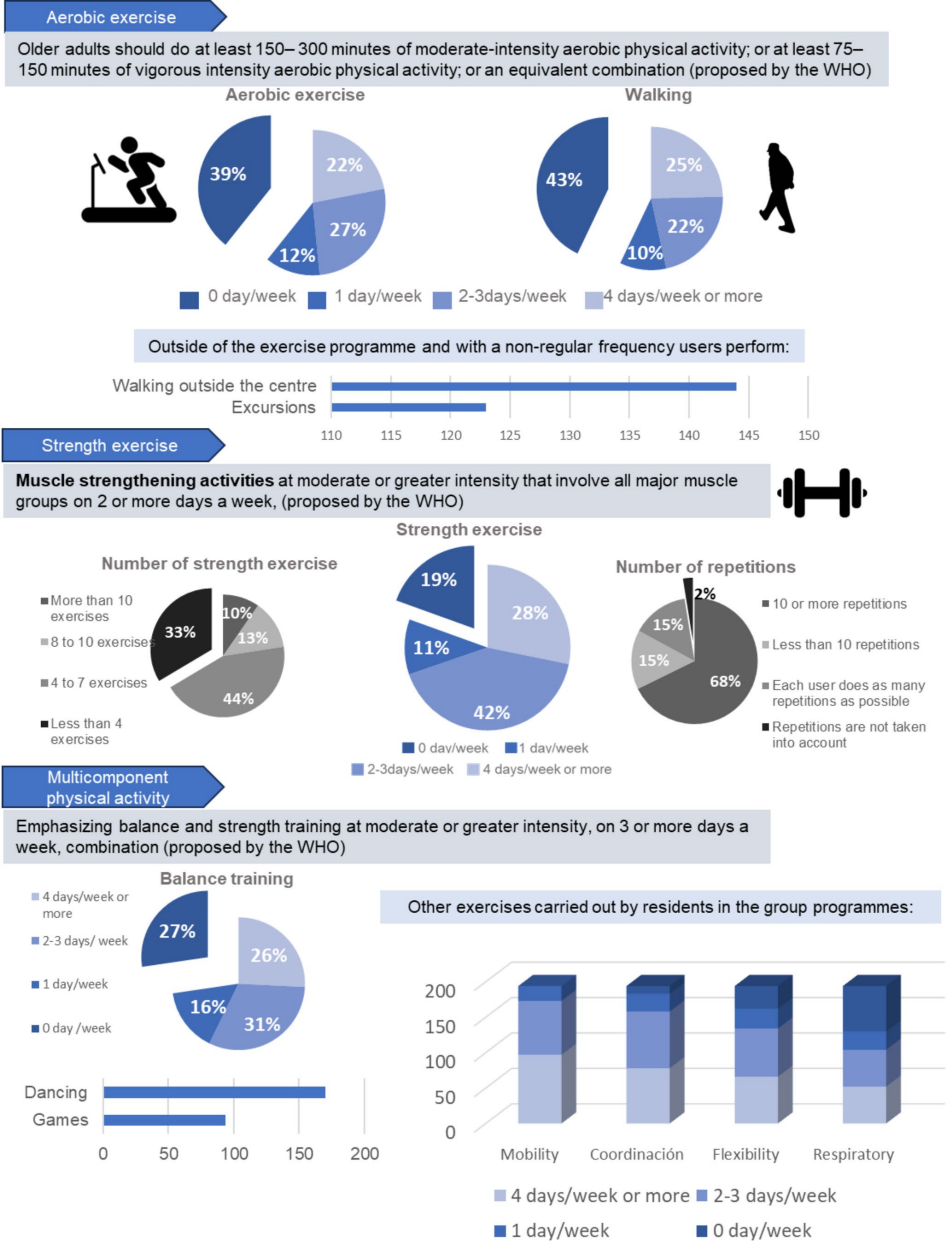
Types of exercise carried out by professionals for institutionalised older persons

Differences were found among the study participants in terms of the implementation of the WHO clinical guidelines according to types of exercises (aerobic exercise, strength exercise and multicomponent physical activity, including balance training) (Fig. 1). Thus, aerobic exercise was performed by 117 (60,3%) subjects of the sample. However, the intensity of the activity and heart rate were only monitored by 17 and 12 participants, respectively. Only 10 participants stated that they performed moderate-intensity exercises, whereas three performed vigorous-intensity exercise. Most of the participants performed aerobic exercise twice a week (27%). As a form of aerobic exercise, many participants (57,7%) reported practicing gait training with their users, often more than four days a week (25%). However, a high percentage of participants (39% and 43%) did not include aerobic exercise (39%). In addition to the aerobic training within the exercise programs, some participants referred going for walks with the residents outside the center (72%) or went on outings (61,5%).

Strength exercises were performed by 81,4% of the respondents, mainly with a frequency of two to three days per week (42%). The number of strength exercises varied according to the respondents, although many claimed to perform between four and seven exercises (44%). However, a high percentage (33%) stated that they performed less than four different strength exercises. Usually, 10 or more repetitions of strengthening exercises were performed (68%). Additionally, 15% of the participants stated that the repetitions depended on the users’ abilities, allowing them to do as many repetitions as possible and 2% of respondents did not consider the number of repetitions.

Within the sample, 27% did not perform balance training exercises. However, 31% stated that they did balance training at least two days a week, and 26% performed it four days a week or more. Furthermore, some participants included activities in their group programs that could challenge balance, such as dancing (50%) and dynamic games (87,6%).

These results highlight that the percentage of professionals who either never did exercise interventions or only scheduled exercise less than twice a week was very high for aerobic exercise (51%), balance (43%), and strengthening (30%).

Besides the exercises recommended in the WHO guidelines, the participants reported including other types of exercise in their programs such as mobility (100%), coordination (95,4%), flexibility (81,5%), or breathing interventions (69,1%). These exercises were performed with varying frequencies, usually two or three days a week.

Differences between the professionals’ working hours and the weekly time spent on group exercises were also identified. As shown in Table 3, no significant differences were observed between each professional group (full time or part time working hours) except for the weekly hours of the exercise program (*p* = 0.035) and weekly strength (*p* = 0.024), balance (*p* = 0,006) and coordination (*p* = 0,030) activities, which was higher in the group of full-time professionals.

**Table 3:** Weekly time dedicated to group exercise program among full-time and part-time professionals

Weekly frequency of…	Full time ≥ 35 h/week (*n* = 121)		Part-time < 35 h/week (*n* = 73)		*p*
	M	SD	M	SD	
Hours of the programme	4,09	2,68	3,32	2,00	**0**,035*
Aerobic exercise	2,17	2,33	1,72	2,04	0,178
Strength exercises	2,88	2,06	2,20	1,93	**0**,024*
Balance exercise	2,57	2,06	1,73	2,02	**0**,006*
Mobility exercises	3,94	1,85	3,61	2,04	0,268
Flexibility exercise	3,00	2,20	2,61	2,20	0,243
Coordination exercise	3,55	1,91	2,89	2,10	**0**,030*
Breathing exercise	2,33	2,12	1,95	2,30	0,254
Walking exercise	1,93	2,00	2,23	2,38	0,849

* *p* < 0.05

### Barriers or limitations identified by professionals regarding using or knowing the WHO guidelines

Over half of professionals (60,5%) identified some limitations for transferring WHO guideline recommendations to the practice of exercise programs. The main barriers identified were the lack of time (79,3%) and the size of the exercise program groups (66,9%), because the professionals felt that the groups were very large. In addition, other barriers such as lack of staff (58,7%) or lack of resources (54,4%). Were also identified. However, generally, the participants did not find their own training a limitation since 67,8% of the respondents claimed to be updated. Data on limitations are shown in Table 4.

**Table 4:** Barriers or limitations identified by professionals in using or knowing the WHO guidelines

Limitations for the application of guidelines	(*N =* 121) (%)
Lack of time	96 (79,3%)
Large groups	81 (66,9%)
Lack of staff	71 (58,7%)
Lack of resources	66 (54,5%)
Lack of spaces	61 (50,4%)
Lack of material	61 (50,4%)
Lack of updating	39 (32,2%)

## Discussion

This study aimed to investigate how the exercise programs for older adults are usually implemented in LTC care institutions in Spain. Also, to estimate to what extent the professionals know and use the WHO recommendations and guidelines for exercising among older people in LTC facilities. And, lastly, to know the limitations identified by health professionals regarding the application of the WHO guidelines in clinical practice.

The results showed that, as shown in the literature [30], exercise programs are generally performed in groups in almost every institution. The centers generally had one or two exercise groups, and these were usually quite large (10–20 people per group). The groups were mainly separated by functional and cognitive ability of the users, which could make it easier to carry out the exercises recommended in the guidelines. However, the groups were mostly monitored by just one professional, which could hinder the compliance of the recommendations in the guidelines and reduce the ability of professionals to tailor exercise to users [24]. Regarding the characteristics of the exercise programs, a significant proportion of the participants claimed to perform the exercise in a seating position whereby some recommendations such as balance exercises or gait training could not be performed. In addition, the time devoted to exercise programs was highly variable, with some centres spending less than three hours per week and others more than six hours per week. This finding appeared to be relevant for the ability to implement the exercises recommended in the WHO guidelines (aerobic exercise, strength exercises, and multicomponent physical activity).

The findings highlight significant gaps between the WHO guidelines and the implementation of exercise programs in LTC facilities. Exercises are often scheduled less than twice a week, moreover, the prevalent use of seated positions indicate potential areas for improvement.

The data also revealed that the recommendations and guidelines were not frequently known and followed by the health professionals when they organized and directed the exercise programs. These results are consistent with the literature [25], as some studies indicate that the implementation of evidence-based recommendations in clinical practice is challenging and not always performed correctly. Our findings show that knowing the specific guidelines leads to greater compliance. However, in contrast to the literature [31], in this study, professional training did not appear relevant in terms of knowledge and implementation of the guidelines.

It should be noted that in this study, the socio-demographic and educational characteristics of the health professionals were not relevant, thus neither supporting or refuting previous studies [32].

Professionals commonly schedule the following types of exercise more than two times a week in group programs: aerobic, strength, balance, mobility, flexibility, coordination, breathing, exercises and gait training [33]. They consider the recommendations on exercise Guidelines to Counteract Physical Deconditioning in LTC facilities [33]. Some of the exercises were also recommended by the WHO guidelines. However, the study participants seemed to include them in different ways during their daily training at the centres. These differences mainly concern the frequency of the sessions, the duration of the sessions, or the intensity applied. For instance, for aerobic exercise, the WHO guidelines recommend older adults to practice at least 150–300 min of moderate-intensity exercise or 75–150 min of vigorous exercise. Nevertheless, a very low number of respondents monitored the users’ heart rate or considered any intensity control system, therefore, despite claiming to perform aerobic exercise, the recommendations were not followed appropriately.

Furthermore, gait training was carried out by a low number of participants; despite the fact that previous studies have shown that gait training is one of the activities that is most demanded by older people as it increases their sense of independence [34–37]. Gait disorders may lead to rapid loss of activities of daily living, and are also related to higher risk of disabilities, falls and mortality. A study that analysed the characteristics of gait in LTC facilities, showed that the gait speed parameters of institutionalized older people placed them at risk of falling and other adverse events [38].

Concerning muscle strength exercises, multiple benefits have been demonstrated in the literature [39], such as the maintenance of normal blood parameters, improvement of cardiovascular functioning, prevention of osteoporosis and sarcopenia and even the prevention of mental disorders, which is why strength exercises are highly recommended in the guidelines. In this sense, a high number of participants in our study reported that this type of exercise was included in their exercise program and, moreover, they performed it at the recommended frequency. In contrast to the low monitoring of aerobic exercise, some respondents considered the number of strength exercises and the number of repetitions when monitoring exercise intensity. This could be because most users performed the exercise in a seated position and strength exercise supervision may be easier to monitor by a single professional compared to aerobic exercise.

The WHO guidelines recommend multicomponent physical activity, which includes balance training. This component was performed by a high number of participants and, apparently, with the recommended frequency. However, as mentioned above, a considerable percentage of residents (25%) carried out the exercise in a seated position and gait training was not performed very frequently. It would be interesting to know how balance training is performed.

Evidence-based practice is essential in the health care setting as it supports the quality of patient care. There are currently several clinical guidelines and recommendations [14, 20, 23] that indicate the recommended amount and types of exercise for each population group, including older people. However, these recommendations are not usually adapted to institutionalized older people [24, 25]. Due to the great heterogeneity of this population, together with comorbidities and decreased independence, the implementation of exercise recommendations is very difficult. Thus, as shown in the results of our study and in previous studies [25, 40], not all health professionals follow these recommendations closely in their daily practice of the exercise programs [40].

Professionals with full-time contracts may have more availability to schedule exercise sessions more frequently compared to those with part-time contracts. The correlation between professional working hours and exercise frequency suggests that increasing staff capacity could enhance program delivery.

In this sense, the participants of our study, similar to other studies [41], found some limitations in updating to new guidelines or in using the guidelines in their clinical practice. According to most participants, as supported by the literature [42], this could be due to lack of time and large exercise groups, among other reasons. These factors could significantly impact the ability of professionals to effectively adhere to the guidelines.

The availability of exercise equipment was associated with greater weekly time spent on certain tasks in Australian residential aged care facilities. This highlights the importance of the availability of equipment and spaces in facilities for quality care for older adults [43].

Related to the lack of implementation of clinical guidelines in institutionalized older people, the results of our study also show a correlation between the hours per week that the professional works and the number of hours spent on the different types of exercises included in the programs. This finding is interesting and could be considered by the management of LTC centers to offer users a better quality of care.

## Limitations of the study

These results should be interpreted considering some limitations. There was a low response rate to the survey. However, previous surveys have also reported low response rates. Therefore, our results may not be representative of all physiotherapists working in LTC institutions. Also, as often occurs in surveys, there is a risk of self-reported bias, and participants’ responses may not be entirely realistic.

The main limitation is that the exercise programming in resident groups was evaluated exclusively through a self-report survey and was not based on resident reports or direct observation of the practice of exercise programs. Although challenging to achieve, linking self-reported exercise activity to residents’ observations and experiences is an area of possible future study. Consequently, in the current study, there may be discrepancies between the exercise plans that exercise professionals plan to implement and the actual execution of these group exercises in LTCs.

## Conclusions

Concerning the characteristics of exercise programs in LTC facilities, our findings denote that they are usually implemented in one or two groups, built on the basis of functional and cognitive abilities, and generally supervised by a single professional. The frequency of exercise depends on the facility, ranging from less than three hours to over six hours per week. Most activities are performed in a sitting position, and they usually include exercise of different typologies: aerobic, strength, balance, mobility, flexibility, breathing or coordination, as well as gait training.

The recommendations included in the WHO guidelines are familiar to many professionals; however, the truth is that many of the recommendations are difficult to implement in group exercise programs organized in LTC facilities.

The main limitations identified for the implementation of WHO guidelines in exercise programs were two: limited time, and large exercise groups. Thus, addressing the aforementioned limitations by increasing the allocated timeframe and optimizing group sizes may help to better align the current exercise programs to the recommendations extolled by the WHO guidelines. Future research should focus on linking self-reported exercise activity with residents’ observations and experiences, to acquire a deeper understanding of the programs’ effectiveness.

The findings stemming from our results should be considered by health professionals working with institutionalized older adults, to enhance the quality of life among residents.

## Data Availability

No datasets were generated or analysed during the current study.
